# The Rat IgGFcγBP and Muc2 C-Terminal Domains and TFF3 in Two Intestinal Mucus Layers Bind Together by Covalent Interaction

**DOI:** 10.1371/journal.pone.0020334

**Published:** 2011-05-23

**Authors:** Hao Yu, Yonghong He, Xin Zhang, Zhihong Peng, Yongtao Yang, Rong Zhu, Jianying Bai, Yin Tian, Xiaohuan Li, Wensheng Chen, Dianchun Fang, Rongquan Wang

**Affiliations:** Department of Gastroenterology, Southwest Hospital, Third Military Medical University, Chongqing, People's Republic of China; University of South Florida College of Medicine, United States of America

## Abstract

**Background:**

The secreted proteins from goblet cells compose the intestinal mucus. The aims of this study were to determine how they exist in two intestinal mucus layers.

**Methodology/Principal Findings:**

The intestinal mucosa was fixed with Carnoy solution and immunostained. Mucus from the loose layer, the firm layer was gently suctioned or scraped, respectively, lysed in SDS sample buffer with or without DTT, then subjected to the western blotting of rTFF3, rIgGFcγBP or rMuc2. The non-reduced or reduced soluble mucus samples in RIPA buffer were co-immunoprecipitated to investigate their possible interactions. Polyclonal antibodies for rTFF3, the rIgGFcγBP C-terminal domain and the rMuc2 C-terminal domain confirmed their localization in the mucus layer and in the mucus collected from the rat intestinal loose layer or firm layer in both western blot and immunoprecipitation experiments. A complex of rTFF3, which was approximately 250 kDa, and a monomer of 6 kDa were present in both layers of the intestinal mucus; rIgGFcγBP was present in the complex (250–280 kDa) under non-reducing conditions, but shifted to 164 kDa under reducing conditions in both of the layers. rMuc2 was found mainly in a complex of 214–270 kDa under non-reducing conditions, but it shifted to 140 kDa under reducing conditions. The co-immunoprecipitation experiments showed that binding occurs among rTFF3, rIgGFcγBP and rMuc2 in the RIPA buffer soluble intestinal mucus. Blocking the covalent interaction by 100 mM DTT in the RIPA buffer soluble intestinal mucus disassociated their binding.

**Conclusions/Significance:**

Rat goblet cell-secreted TFF3, IgGFcγBP and Muc2, existing in the two intestinal mucus layers, are bound together by covalent interactions in the soluble fraction of intestinal mucus and form heteropolymers to be one of the biochemical mechanisms of composing the net-like structure of mucus.

## Introduction

The gastrointestinal tract is the largest and most exposed surface in humans and other mammals, and thus, the epithelium is vulnerable and needs to be protected. The mucous barrier over the epithelium is important but is often ignored as a research topic. In particular, an extensive amount of bacteria inhabit the lumen within the small intestine and colon and live in a mutual relationship with the host. [1], [2] The disruption of the lumenal barrier is a feature of common and important gastrointestinal disorders, which include inflammatory bowel diseases (IBDs). [3], [4], [5]

The colonic mucus is composed of two different layers: the outer layer is the loose layer and is easy to remove, and the inner layer is a firm layer, which is strongly attached to the epithelium and must be removed by scraping. [6] A recent study has revealed that the inner layer of mucus is devoid of bacteria and that the bacteria only reside within the loose layer of mucus. [7] Mucus is a highly viscous and elastic barrier that protects the mucosal surfaces by selectively trapping and shedding pathogens, toxins, and ultrafine particles, while allowing the rapid flux of nutrients, antibodies, and cells of the mucosal immune system.

Mucin 2 (MUC2), trefoil factor 3 (TFF3) and IgGFc gamma binding protein (IgGFcγBP) are the main proteins secreted from goblet cells. TFF3 has been implicated in multiple mucosal protection and repair processes, where it plays a key role in the integrity of the mucous epithelia. [8] It is clear that only the luminal application, but not the systemic delivery, of recombinant TFF3, is protective in colitis models; [9] however, its exact mechanism is unclear, and it is consistent with the lack of characterization of specific receptor of TFF3, to date. [10] It is important to understand the biochemical role of TFF3 in the maintenance of the intestinal mucus, although the existing reports are controversial. Hansson et al. reported that TFF3 was not found in both of the intestinal mucus layers in their proteomic study, [11] but human TFF3 does form disulfide-linked heterodimers with the mucus-associated FCGBP protein, and the intestinal mucus was shown to be a reservoir for TFF3. [12] Therefore, it is necessary to explore the potential role of TFF3 in maintaining the viscosity and elasticity of the intestinal mucus.

The IgGFcγBP protein is a mucin that has received little attention; it binds only to the Fc portion of IgG, not to IgG Fab, IgA, or IgM. [13] It has been suggested that the human IgGFcγBP protein is secreted from goblet cells and that some of the IgGFcγBP protein can be secreted into the circulatory system by an unknown mechanism in patients with autoimmune disease. [14] We previously reported that the rat IgGFcγBP protein was depleted during dextran sulfate sodium (DSS)-induced colitis. [15] Furthermore, proteomic analyses of the intestinal mucus revealed that the IgGFcγBP protein was one of the important components of mucus and that there was an interaction in the insoluble, firm layer mucus between the human IgGFcγBP protein and MUC2 [11] as well as between the IgGFcγBP protein and TFF3. [12] Thus, it is necessary to explore how the IgGFcγBP protein takes part in the formation and organization of the mucus network as well as its inherent function.

MUC2 is the predominant mucin expressed in the intestine of humans, rats and mice, and MUC2 mucin monomers form dimers in the endoplasmic reticulum via intermolecular disulfide bonds between its carboxy (C)-terminal cysteine knot domains. [16], [17] The MUC2 mucin is the major structural component of the colonic mucus gel. Their N- and C-terminal cysteine-rich domains are involved in mucin polymerization. The *Entamoeba histolytica* cysteine proteases can cleave the C-terminal domain of MUC2 to disrupt the protective mucus barrier. [18] The mechanical properties of biopolymer networks of the intestinal mucus are helpful to understand its physiological significance and the pathophysiological involvement in a series of bowel diseases.

Based on polyclonal antibodies against rat TFF3, the IgGFcγBP C-terminal domain and the Muc2 C-terminal domain, in this report, we investigated the distribution of rTFF3, rIgGFcγBP and rMuc2 in the two intestinal mucus layers and the mucosa, their existing molecular patterns (especially in the two mucus layers) under reduced or non-reduced conditions, and their potential binding in the mucus layer.

## Results

### The collection of the mucus from the loose or firm layers of rat intestine

**Figure 1:**
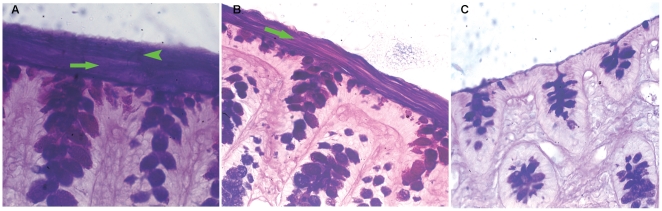
Micrographs of rat intestinal mucosa of the colon fixed with Carnoy solution and stained by AB/PAS staining method (original magnification ×400). Normal colonic mucosa contained two mucus layers, the top, loose layer of mucus (arrowhead) and a stratified, firm layer of mucus (arrow) (A); the colonic mucosa after suction of the mucus contained only the stratified firm layer of mucus (arrow) (B); the colonic mucosa after suction and scraping of the mucus (C).

The intact mucosa with two mucus layers, the suctioned mucosa with only firm mucus layer and the scraped mucosa without two mucus layers were fixed using Carnoy solution and stained by Alcian blue-periodic acid Schiff (AB/PAS) method. As shown in Figure 1A, there were two mucus layers above the intestinal epithelium: the top layer (arrowhead) of mucus appeared loose, and the low layer (arrow) of mucus appeared to be lamellar. After suction of the loose layer of mucus, the firm layer remained (Figure 1B); the intestinal mucosa without the firm layer of mucus was left after scraping of firm mucus layer (Figure 1C). Thus, the collection method of the loose layer or firm layer of the intestinal mucus was facilitated for the following experiments.

### The presence of rTFF3, rIgGFcγBP and rMuc2 in the mucus layers by immunohistochemistry

**Figure 2:**
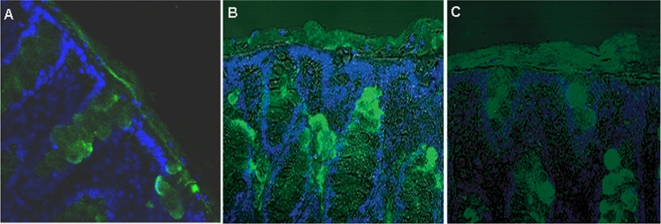
Immunohistochemical staining of the rat intestinal mucosa fixed with Carnoy solution (original magnification ×400). Rat TFF3 (A), IgGFcγBP (B) and Muc2 (C) existed in the two mucus layers and goblet cells.

To confirm their presence of rTFF3, rIgGFcγBP and rMuc2 at the mucus layer and to determine any difference in their distribution between the loose and firm layers, an immunohistochemical staining of the rat intestinal mucosa, fixed with the Carnoy solution, was carried out with anti-rTFF3, anti-rIgGFcγBP and anti-rMuc2 polyclonal antibodies. As shown in Figures 2A, rTFF3 was present in the two intestinal mucus layers and the goblet cells within the mucosa. The immunostaining of rIgGFcγBP (Figures 2B) and rMuc2 (Figures 2C) revealed that both were present in the goblet cells within the mucosa and two intestinal mucus layers. They were distributed evenly in the loose and firm layers, with no significant difference between them.

### rTFF3 in the loose and firm mucus layers and the mucosa without the two layers under reduced and non-reduced conditions

**Figure 3:**
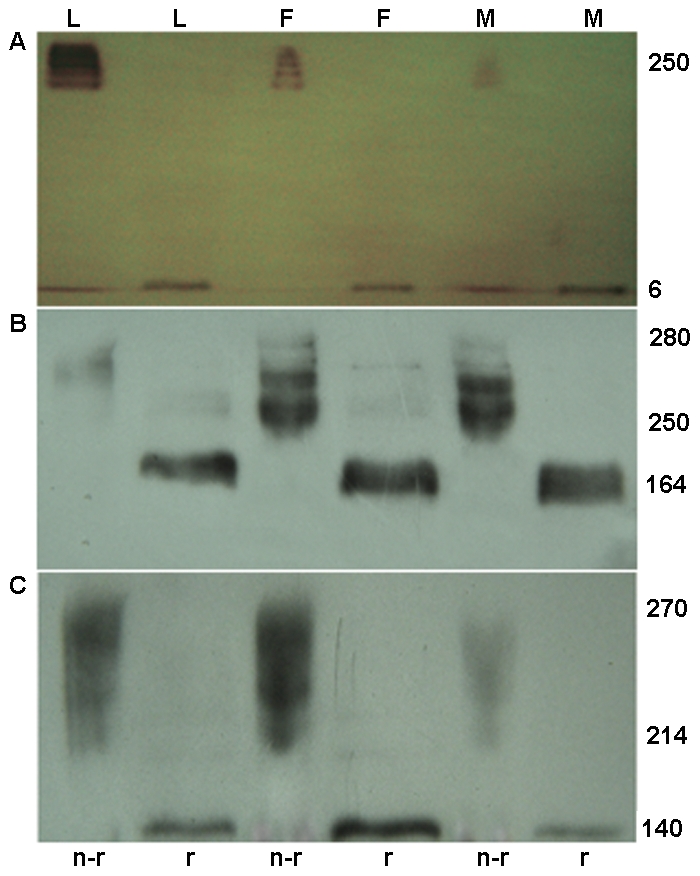
Rat TFF3 (panel A), IgGFcγBP (panel B) and Muc2 (panel C) in the loose (L) and firm (F) mucus layers and mucosa free of mucus (M) under reduced (r) and non-reduced (n-r) conditions.

To discriminate a possible difference in the rTFF3 distribution between the loose and firm mucus layers and the mucosa without two mucus layers, we investigated the presence of rTFF3 in the mucus from the loose or firm mucus layers and the mucosa without the two mucus layers under reduced and non-reduced conditions using sodium dodecyl sulfate polyacrylamide gel electrophoresis (SDS-PAGE) and western blotting. As shown in panel A of Figure 3, under non-reducing conditions, rTFF3 was present in the loose layer mucus, mainly in the form of complex with a molecular weight of 250 kDa, and a small amount was present, presumably as a monomer, with a molecular weight of 6 kDa (lane 1); under reducing conditions, all of the rTFF3 identified was at a molecular weight of 6 kDa (lane 2). Similarly, rTFF3 was present in the firm layer mucus and mucosa without two mucus layers, where it was found mainly in the form of a complex (250 kDa) and a small amount of monomer (6 kDa) under the non-reduced conditions (lanes 3 and 5) and a monomer (6 kDa) under the reduced conditions (lanes 4 and 6). Although the immunoreactivity of rTFF3 among the loose mucus layer, firm mucus layer and mucosa without two mucus layers was not same, the molecular pattern of rTFF3 among them was similar. This finding indicates that rTFF3 was complexed with an unknown protein(s) in the goblet cells within the mucosa and that this complex was secreted into the firm mucus layer until the formation of the loose mucus layer.

### rIgGFcγBP in the loose and firm mucus layers and the mucosa without the two layers under reduced and non-reduced conditions

To discriminate the possible difference of rIgGFcγBP distribution between the loose mucus layer, firm mucus layer and mucosa without two mucus layers, the presence of rIgGFcγBP was determined in the mucus from the loose and firm mucus layers and the mucosa without the two mucus layers under reducing and non-reducing conditions using SDS-PAGE and western blotting. As shown in panel B of Figure 3, rIgGFcγBP was present in the loose layer mucus, mainly in the form of a complex with a molecular weight of 250–280 kDa under the non-reducing conditions (lane 1); however, under reducing conditions, rIgGFcγBP was mainly present at a molecular weight of 164 kDa (lane 2). As with the loose mucus layer, rIgGFcγBP was also present in the firm layer mucus and the mucosa without the two mucus layers, mainly in the form of a complex (250–280 kDa) under non-reduced conditions (lanes 3 and 5), and rIgGFcγBP existed as a monomer (164 kDa) under reduced conditions (lanes 4 and 6). This results indicate that rIgGFcγBP was complexed with an unknown protein(s) in the goblet cells within the mucosa and that this complex was secreted into the firm mucus layer until the formation of the loose mucus layer.

### rMuc2 in the loose and firm mucus layers and the mucosa without the two layers under reduced and non-reduced conditions

To discriminate the possible difference of rMuc2 distribution between the loose mucus layer, firm mucus layer and mucosa without two mucus layers, the presence of rIgGFcγBP was determined in the mucus from the loose and firm mucus layers and the mucosa without the two mucus layers under reducing and non-reducing conditions using SDS-PAGE and western blotting. As shown in panel C of Figure 3, under non-reducing conditions, rMuc2 was present in the loose layer mucus, mainly in the form of a complex with a molecular weight of 214–270 kDa (lane 1), whereas under reducing conditions, rMuc2 was mainly present at a molecular weight of 140 kDa (lane 2). rMuc2 was also present in the firm layer mucus and mucosa without two mucus layers, mainly in the form of a complex (more than 214 kDa) under non-reducing conditions (lanes 3 and 5). We found that rMuc2 existed as a monomer (140 kDa) under reducing conditions (lanes 4 and 6). As with the other two proteins, this finding indicates that rMuc2 was complexed with an unknown protein(s) in the goblet cells within the mucosa and that this complex was secreted into the firm mucus layer until the formation of the loose mucus layer.

### The immunoprecipitation of rTFF3, rIgGFcγBP or rMuc2 with anti-rTFF3, anti-rIgGFcγBP or anti-rMuc2 polyclonal antibodies in the intestinal mucus

**Figure 4:**
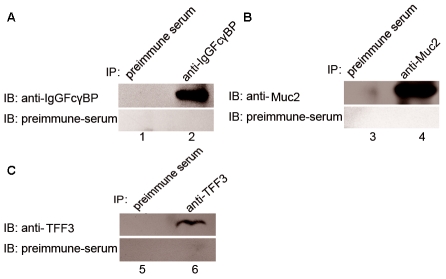
Immunoprecipitation of rat TFF3 (panel C), IgGFcγBP (panel A) or Muc2 (panel B) in the intestinal mucus with the corresponding antibodies. 6 kDa, 164 kDa or 140 kDa proteins were identified with their respective antibodies, but not with their preimmune serum, in the reduced intestinal mucus dissolved in RIPA buffer.

To confirm the specificity of the anti-rTFF3, anti-rIgGFcγBP or anti-rMuc2 polyclonal antibodies used in the following immunoprecipitation experiment, we used the anti-rTFF3, anti-rIgGFcγBP or anti-rMuc2 polyclonal antibodies to immunoprecipitate the reduced intestinal mucus, which included the loose layer and firm layer. As shown in Figure 4, when the reduced mucus was immunoprecipitated with anti-rIgGFcγBP polyclonal serum or preimmune serum, and detected with anti-rIgGFcγBP polyclonal serum or preimmune serum, a 164 kDa polypeptide could be detected with the anti-rIgGFcγBP polyclonal serum in the mucus immunoprecipitated by the anti-rIgGFcγBP polyclonal serum, but not in the mucus immunoprecipitated by the preimmune serum. However, there was no positive polypeptide found in the mucus immunoprecipitated by preimmune serum after blotting with either the anti-rIgGFcγBP polyclonal serum or preimmune serum. This result indicates that the anti-rIgGFcγBP polyclonal serum produced in the study was suitable for the immunoprecipitation experiment. The same experiment using anti-TFF3 or anti-Muc2 polyclonal serum also confirmed that both of these polyclonal antibodies were suitable for the immunoprecipitation experiment.

### The confirmation of the binding of rTFF3, rIgGFcγBP and rMuc2 in the intestinal mucus by a co-immunoprecipitation experiment

**Figure 5:**
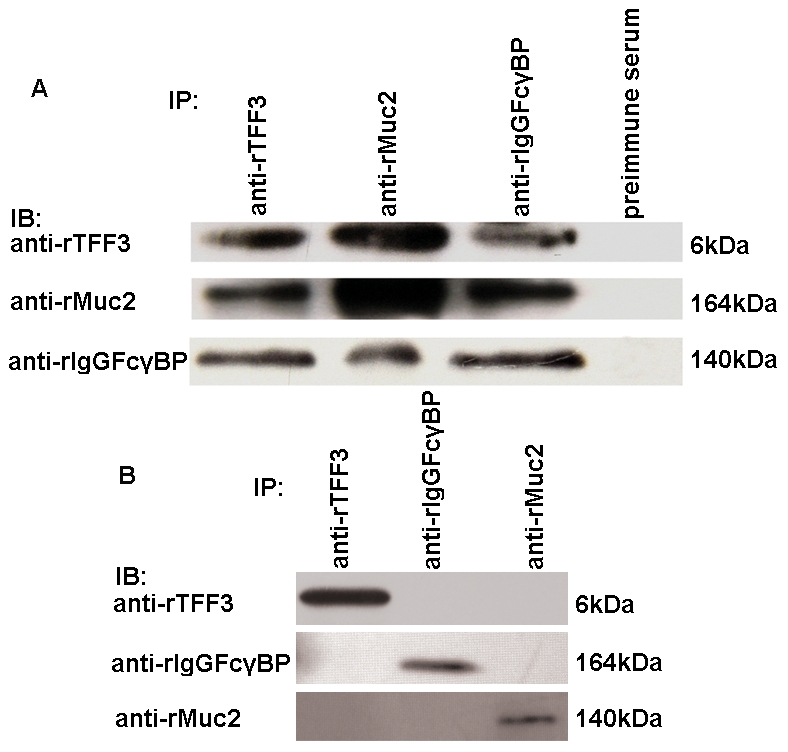
Coimmunoprecipitation of rat TFF3, IgGFcγBP or Muc2 in the non-reduced (A) and reduced (B) intestinal mucus and blotted with each antibody. 6 kDa proteins were detected with the anti-rTFF3 antibody in the immunoprecipitates with anti-rTFF3, anti-rIgGFcγBP and anti-Muc2 antibodies. 164 kDa proteins were detected with the anti-rIgGFcγBP antibody in the immunoprecipitates with anti-rTFF3, anti-rIgGFcγBP and anti-rMuc2 antibodies. The 140 kDa proteins were detected with the anti-rMuc2 antibody in the immunoprecipitates with anti-rTFF3, anti-rIgGFcγBP and anti-rMuc2 antibodies.

The non-reduced mucus from rat intestine was collected from the fresh intestine of the sacrificed rat and contained not only the loose layer but also the firm layer; the sample was immunoprecipitated with anti-rTFF3, anti-rIgGFcγBP or anti-rMuc2 polyclonal antibodies, separated by SDS-PAGE and detected by anti-rTFF3, anti-rIgGFcγBP or anti-rMuc2 polyclonal antibodies. As shown in Figure 5A, in the immunoprecipitates using the anti-TFF3 polyclonal antibody, a 6 kDa product was detected using anti-rTFF3, anti-rIgGFcγBP and anti-rMuc2 polyclonal antibodies, but it was not detected in the immunoprecipitates using pre-immune serum. In the immunoprecipitates using the anti-rIgGFcγBP polyclonal antibody, a 164 kDa product was detected using the anti-rTFF3, anti-rIgGFcγBP and anti-rMuc2 polyclonal antibodies, but the result was negative in the immunoprecipitates using pre-immune serum. In the immunoprecipitates using the anti-rMuc2 polyclonal antibody, a 164 kDa product was detected with the anti-rTFF3, anti-rIgGFcγBP and anti-rMuc2 polyclonal antibodies, but no such product was detected in the immunoprecipitates using pre-immune serum. Thus, the results indicate that rTFF3, rIgGFcγBP and rMuc2 in the rodent intestinal mucus were associated with each other when the sample was processed under the non-reducing conditions.

### The disruption of covalent interactions by dithiothreitol (DTT) disassociates the binding among rTFF3, rIgGFcγBP and rMuc2

The fresh mucus collected from rat intestine containing the loose layer and firm layer were dissolved in RIPA lysis buffer (RIPA) with 100 mM DTT, and the reduced sample was immunoprecipitated with anti-rTFF3, anti-rIgGFcγBP or anti-rMuc2 polyclonal antibodies, separated by SDS-PAGE and detected by anti-rTFF3, anti-rIgGFcγBP and anti-rMuc2 polyclonal antibodies. As shown in Figure 5B, in the immunoprecipitates using the anti-TFF3 polyclonal antibody, a 6 kDa product was detected using only the anti-rTFF3 polyclonal antibody, but not detected using either anti-rIgGFcγBP or anti-rMuc2 polyclonal antibodies. In the immunoprecipitates using the anti-rMuc2 polyclonal antibody, a 140 kDa product was detected only using the anti-rMuc2 polyclonal antibody, but not detected using either anti-rTFF3 or anti-rIgGFcγBP polyclonal antibodies. In the immunoprecipitates using the anti-rIgGFcγBP polyclonal antibody, a 164 kDa product was detected only using the anti-rIgGFcγBP polyclonal antibody, but not detected using either anti-rTFF3 or anti-rMuc2 polyclonal antibodies. This result suggests that the interactions among rTFF3, rIgGFcγBP and rMuc2 in the rodent intestinal mucus are based on covalent bonds, which were disrupted by DTT.

## Discussion

It has long been known that the intestinal mucus layers of the colon are an important defense barrier against bacteria and other toxic factors. Researchers have begun exploring these interactions to gain a deeper understanding of the organization of the mucus layer and the molecular details of its main components. [19] Indeed, a better insight into the biochemical nature of the mucus layer of colon is fundamental for an understanding of how this protective function is achieved. The groups of Holm and Hansson have identified a method for measuring the thickness of the two mucus layers, and they have performed a detailed proteomic analysis of the two colonic mucus layers. [6], [20] MUC2 and IgGFcγBP were identified as two of the main proteins present in the two mucus layers. [11] Although a binding between human MUC2 and IgGFcγBP and human TFF3 and IgGFcγBP in the intestinal mucus layers was determined based on proteomic analyses. [11], [12] It has not been confirmed by other methods, including co-immunoprecipitation experiments, until now.

Rat IgGFcγBP is composed of 2583 amino acids (NM_001164657.1), in which there are 5 potential autoproteolytic cleavage sites (GDPH) [21], [22] and the 11 tandem repeats. The epitope of CGKIRDPKGPFATC, which was recognized by the anti-rIgGFcγBP antibody, is located in the C-terminal region of rIgGFcγBP. If proteolysis occurs at the last predicted GDPH site, the resulting C-terminal domain of IgGFcγBP would be 926 residues long. We have confirmed previously that it was depleted in the animal model of DSS-induced colitis and expressed in the epithelium of immature intestinal mucosa, [15] and that it is distributed in various mucin-producing cells, is present in body fluids, and can be found in the serum of some autoimmune diseases. [13], [23] Although IgGFcγBP can bind to the Fc portion of IgG, the biological function of rat IgGFcγBP has remained unclear, due to the lack of a potential antibody against rat IgGFcγBP. The presence of the sequence of repetitive vWD domains may indicate additional functions, as suggested for the molecules of other mucins, in which the vWD domains are involved in oligomerization. Our present data provided a direct evidence for the covalent interaction of the C-terminal domain of rat IgGFcγBP with both the C-terminal domain of rMuc2 and TFF3.

The facilitation of the homodimer formation via the C-terminal domain of rMuc2 has been previously reported [16] In the present study, we confirmed that the C-terminal domain of rMuc2 can form a heteropolymer with the C-terminal domain of rat IgGFcγBP and rTFF3 in the soluble mucus through covalent interactions. In agreement with the findings by Hansson's group, the human MUC2 and mouse Muc2 bound to the N-terminal regions of IgGFcγBP in the insoluble mucus via covalent interaction. Whereas the N-terminal regions of IgGFcγBP bind to human MUC2 and mouse Muc2, the C-terminal domain of IgGFcγBP binds to rat Muc2 and rat TFF3; it is conceivable that the heteropolymers formed might be responsible for the nature of the net-like, disulfide bond-based structures of the intestinal mucus. It is clear that the rigid structure of the intestinal mucus in the firm layer of mucus provides an important barrier, preventing the infiltration of bacteria, and that the loose layer is the result of the inflation of the firm mucus layer when there is a large load of bacteria. [24]

In the report of Hansson, their proteomic analysis did not include human or mouse TFF3 in the two intestinal mucous layers. TFF3 possesses a broad range of activity, including the modification of epithelial growth, cell motility, and acid secretion. [25], [26] However, there is a lack of identification of their putative receptors. Whether TFF3 exerts a potential role in maintaining the net-like, disulfide bond-based structures of the intestinal mucus is not well understood. Hoffmann's group has isolated TFF3 from human colonic extracts under non-reducing conditions and purified a TFF3 heterodimer; after characterization by LC-ESI-MS/MS analysis, they confirmed that TFF3 bound to IgGFcγBP via a disulfide bond, which could be disrupted by hydrogen sulfide. [12]

The major result of our present study indicated that rat TFF3, the rat Muc2 C-terminal domain and the rat IgGFcγBP C-terminal domain undergo disulfide bond-based interactions in the RIPA buffer soluble fragments of two mucous layers and suggesting one of the biochemical basis of the net-like structures of intestinal mucus. The data provide a better insight into the biochemical nature and organization of the rat intestinal mucus layer, which will be fundamental for an understanding of how this protective function of the rat intestinal mucus layer is constructed and of how this mechanism might relate to the pathophysiology of intestinal diseases, such as IBD, infection of the intestine and intestinal carcinoma. [27] Although GuHCl extraction of the intestinal mucus is the traditional method to dissociate non-covalent bonds and maintain covalent interactions, the lysis of the intestinal mucus using RIPA buffer can also maintain the covalent interaction between molecules and is suitable for the detection of interactions between proteins.

The possible biological functions of the heteropolymer among the rTFF3-rMuc2 C-terminal domain-rIgGFcγBP C-terminal domain for the structure of the rat intestinal mucus are unknown. rMuc2 and rIgGFcγBP are two major components of the intestinal mucus and provide the characteristics of the intestinal mucus barrier. rTFF3 might exert an extra function to strengthen the intestinal mucus barrier.

The weakness of present study is that we only confirmed the binding among the rTFF3-rMuc2 C-terminal domain-rIgGFcγBP C-terminal domain in the RIPA buffer soluble fraction of rat intestinal mucus, in which represents a small amount of the intestinal mucus components. It is still unclear whether there is a similar binding among the rTFF3-rMuc2 C-terminal domain-rIgGFcγBP C-terminal domain in the insoluble fraction of rat intestinal mucus due to the technical limitations of immunoprecipitation experiments.

## Materials and Methods

### Materials

Eight-week-old, specified pathogen-free Wistar rats (purchased from the Animal Facility, Third Military Medical University, Chongqing, China) were housed at a constant temperature and humidity on a 12-hour light/dark cycle. One week before and during the experiment, the rats were housed individually. The rats had free access to a standard pelleted diet and sterilized tap water. All experiments were performed with the approval of the Animal Studies Ethics Committee of Southwest Hospital, Third Military Medical University(permit number: sw20070612).

### The anti-rTFF3, anti-rIgGFcγBP or anti-rMuc2 polyclonal antibodies

The produce and characterization of the anti-rTFF3, anti-rIgGFcγBP or anti-rMuc2 polyclonal antibodies was described previously. [15], [16], [28], [29], [30], [31], [32] The anti-rMuc2 polyclonal antibody was raised using ‘d-link’, a deglycosylated 118 kDa glycoprotein in New Zealand rabbits and the anti-rMuc2 polyclonal antibody was strongly reactive to the synthetic peptide, DEWLVNDPSKPHC. The anti-rTFF3 polyclonal antibody was raised using a 21-residue peptide-keyhole limpet hemocyanin (KLH) (QEFVGLSPSQCMAPTNNRVDC) and the anti-rIgGFcγBP polyclonal antibody was raised using a 14-residue peptide (CGKIRDPKGPFATC)-KLH.

### Immunostaining

Segments of the distal rat colon were fixed in water-free Methanol-Carnoy's fixative (60% methanol, 30% chloroform and 10% acetic acid). The tissues were washed in methanol before being embedded in paraffin and sectioned (5 mm). The sections were dewaxed using Xylene Substitute and then hydrated. The antigens were retrieved by microwave heating in 0.01 M citric buffer (pH 6), and the sections were stained with anti-rTFF3 (1∶100), anti-rIgGFcγBP (1∶100) or anti-rMuc2 (1∶200) polyclonal antibodies. FITC-conjugated goat anti-rabbit immunoglobulin was used as secondary antibodies (Boster Biological Technology Co. LTD., Wuhan, P. R. of China), and the DNA was stained with DAPI (Invitrogen, Carlsbad, CA, USA). The images were obtained using a BX51 (Olympus, Tokyo, Japan) fluorescence microscope. The negative controls, using unrelated anti-serum or PBS buffer for the first antibodies, were used to determine the specificity.

### Histological staining

A combined method, utilizing the properties of both the PAS and Alcian blue methods, to demonstrate that the full complement of tissue proteoglycans was employed. Last, the nuclei were stained with hematoxylin for 1 minute, and the sections were dehydrated, cleared and mounted.

### Collection of the mucus from the loose layer or firm layer of rat intestine

Wistar rats from the Animal Facility, Third Military Medical University, Chongqing, China, were fasted for 24 hours before euthanasia. The colon was separated and dissected between the cecum, which was 5 cm distant from the beginning of cecum, and the rectum, which was 2 cm distant from the end of rectum, and cut longitudinally and immediately spread on a smooth and stiff cardboard; the mucus from a dissected 1.5 cm rat colon was collected by suction (loosely adherent). After suction, the mucus from the firm layer of the intestinal mucus layer was scraped (firmly adherent). The remaining mucosa, free of the mucus, was scraped and homogenized for further experiments. A complete, EDTA-free protease inhibitor (Roche Molecular Diagnostics, 4300 Hacienda Drive, Pleasanton, CA 94588 USA) was added to the samples of mucus from the loose layer, firm layer and mucosa (free of mucus).

### SDS-PAGE and western blotting analysis

The collected samples of the loose-layer mucus, firm-layer mucus or homogenized intestinal mucosa, free of mucus, were lysed in SDS sample buffer. The samples were divided, and one was reduced in sample buffer with 100 Mm DTT, while the other was not reduced (as a control); the samples were subjected to SDS-PAGE (12%) after heating for 5 minutes in boiling water. The proteins were then transferred to an Immobilon P membrane. The membranes were blocked for 1 hour in 5% nonfat dry milk in TBST (TBS containing 0.05% Tween-20) and hybridized at 4°C overnight in TBST with the primary antibodies (anti-rTFF3, 1∶500, anti-rIgGFcγBP, 1∶800, or anti-rMuc2, 1∶3000). The membranes were washed three times in PBST, for 5 min each time, and the secondary detection was done using 1∶12,000 dilutions of HRP-conjugated goat anti-rabbit antibody. The membranes were then washed three times, for 10 min each. The blots were processed either with the SuperSignal® West Pico Chemiluminescent Substrate, and the signal was detected by exposing X-ray films to the processed blots. SeeBlue® Plus2 was used as the reference sample of molecular mass standards.

### Immunoprecipitation of rTFF3, rIgGFcγBP or rMuc2 in the intestinal mucus

The freshly collected rat intestinal mucus, including the loose layer and firm layer, was lysed in 5 volumes of RIPA buffer, and a complete, EDTA-free protease inhibitor (Roche) was added. After mixing for 2 hours at 4°C, the samples were centrifuged at 10000**×**g for 10 minutes, and the soluble supernatant was collected and precleared with 50 µl protein-G agarose. But the insoluble pellet which contained a large proportion of the Muc2 mucus was discarded, as the insoluble pellet was not suitable for the immunoprecipitation experiment. The specific proteins were immunoprecipitated during a 16 h incubation at 4°C with anti-rTFF3, anti-rIgGFcγBP, anti-rMuc2 serum or their preimmune sera in the presence of 50 µl protein-G agarose. The immunoprecipitates were washed twice in RIPA buffer (50 mM Tris/HCl [pH 7.5], 150 mM NaCl, 1% (v/v) Nonidet P40 (NP40), 0.5% Sodium deoxycholate, 0.1% SDS and protease inhibitor) and once in 10 mM Tris/HCl buffer (pH 7.4) containing 0.1% NP40 and then were subjected to SDS/PAGE after reduction with 100 mM DTT; then, the proteins were transferred to an Immobilon P membrane, the separated proteins were blotted with anti rTFF3, anti-rIgGFcγBP, anti-rMuc2 serum or their preimmune serum. The further experiment was performed as described previously.

### Co-immunoprecipitation experiment

The non-reduced mucus from the rat intestine was immunoprecipitated with anti-rTFF3, anti-rIgGFcγBP, or anti-rMuc2 polyclonal antibodies or preimmune serum, separated by SDS/PAGE after the immunoprecipitates were reduced with 100 mM DTT and detected by anti-rTFF3, anti-rIgGFcγBP or anti-rMuc2 polyclonal antibodies. To block the possible covalent binding among rTFF3, rIgGFcγBP and rMuc2, the fresh rat intestinal mucus was pretreated with 100 mM DTT; the co-immunoprecipitation and blotting experiment was performed as described above.
